# P23 Advance market commitment for point of care diagnostic focused on neonatal sepsis: estimating costs and effectiveness

**DOI:** 10.1093/jacamr/dlae136.027

**Published:** 2024-08-23

**Authors:** Akhil Bansal, Adam Howes, Ilona Arih

**Affiliations:** Royal Free Hospital, London, UK; London School of Hygiene and Tropical Medicine, London, UK; Royal Free Hospital, London, UK

## Abstract

**Background:**

Antimicrobial resistance (AMR) is a pressing global health concern, responsible for millions of deaths every year. Overuse and misuse of antimicrobials is a significant driver of AMR and is partly driven by a lack of decision-relevant and timely diagnostic tests to guide treatment. Point of care tests (POCT) would be of great public health benefit for neonatal sepsis, which refers to systemic life-threatening infections in newborns in their first 28 days of life. Neonatal sepsis, for which over- and inappropriate prescription of antibiotics are very high, affects 1.3 to 3.9 million neonates and accounts for nearly a quarter of all neonatal deaths. However, due to insufficient market incentives and revenue guarantee for developers, research and development pipelines are stagnant and no such POCT exists. One proposed solution is an advanced market commitment (AMC), a form of ‘pull incentive’ that would aim to ‘pull’ a new POCT to market. In an AMC, donors commit to a fund from which a specified subsidy is paid per unit purchased by low income countries until the fund is exhausted. This aims to strengthen suppliers’ incentives to invest in research, development and capacity.

**Methods:**

This study modelled the costs and outcomes of an AMC for a neonatal sepsis POCT that would seek to identify the presence or absence of sepsis. Modelled for the Indian public healthcare system, the test was conceived as an adjunct to clinical judgement where there is diagnostic uncertainty if a neonate has sepsis. The POCT was compared with current best practice. Benefits of increased identification of neonatal sepsis and decreased burden of AMR were considered; cost of diagnostic tests and antibiotic treatment were considered. Cost-effectiveness and incremental cost-effectiveness were calculated in cost per disability adjusted life year (DALY), from both the perspective of the Indian healthcare system and investors to an AMC.

**Results:**

From our cost-effectiveness model, a POCT would have an incremental cost-effectiveness of $93.56/DALY (compared with current clinical practice) for the Indian healthcare system, with a cost-effectiveness of $5.9/DALY for donors to an AMC. On sensitivity analysis, the cost benefit analysis is contingent on a test with sufficiently high specificity and sensitivity and varies depending on estimated AMR burden.

**Conclusions:**

Although a novel market incentive with some uncertainty in model parameters, an AMC for a neonatal sepsis POCT may be highly effective and cost-effective from both a global health and AMR perspective.

